# Letter to the editor: Use of integrated genomic surveillance by local public health authorities: recommendations based on a mixed-methods study of current adoption, applications and success factors, Germany, 2023

**DOI:** 10.2807/1560-7917.ES.2025.30.28.2500436

**Published:** 2025-07-17

**Authors:** Tim Eckmanns, Sascha Al Dahouk, Benjamin Tittmann, Nadine Litzba, Hanna Buck, Katrin Kremer-Flach, Anke Kohlenberg, Guido Werner, Hendrik Wilking, Sebastian Haller, Torsten Semmler

**Affiliations:** 1 Robert Koch Institute, Berlin, Germany; 2 European Centre for Disease Prevention and Control, Stockholm, Sweden

**Keywords:** Genomics, Surveillance, Epidemiology, Public Health

To the editor: With great interest, we read the article by Bludau et al. ‘*Use of integrated genomic surveillance by local public health authorities: recommendations based on a mixed-methods study of current adoption, applications and success factors, Germany, 2023*’ [1]. The authors make a timely and valuable contribution to the evolving field of genomic pathogen surveillance in Germany. In particular, their practical recommendations – such as prioritising flexibility and scalability, defining application scenarios and objectives, improving data interpretation and analysis capabilities, developing supportive infrastructures, and securing sustainable funding – offer a valuable roadmap for local public health authorities (LPHAs).

In our letter, we aim to expand the ongoing discussion on integrated genomic surveillance (IGS) by offering further perspectives. Given the current structure of the German public health service, which consists of 376 LPHAs and 16 state-level agencies, there is clearly a need for effective federal coordination and cooperation, as well as a common data repository. This is also a prerequisite for feeding IGS data into international surveillance systems.

Bacteria and viruses are known to spread across regions, countries and continents as a result of human mobility, patient transfers and the trade of animals, food and other goods. This leads to outbreaks on national and international scales. Pooling genomic data from diverse regions and countries enables early detection of emerging pathogens as well as resistance and virulence genes, as demonstrated by recent investigations at the European level [2]. Local outbreaks often represent satellite events of broader national or international transmission dynamics. Identifying interregional links and transmission chains facilitates the detection of common transmission routes and infection sources, thereby enabling the rapid containment of outbreaks at all levels of infection control management.

The Robert Koch Institute (RKI), as Germany’s national public health institute, has developed a comprehensive strategy for integrated genomic surveillance of a broad spectrum of notifiable infectious disease pathogens. Data generated through this approach, combined with clinical and epidemiological information, will be incorporated into the national reporting system for infection control and made available for public health purposes [3]. These data will also be transmitted to EpiPulse, supporting the European Centre for Disease Prevention and Control's (ECDC) surveillance and information capacities, in accordance with Regulation (EU) 2022/2371 of the European Parliament and of the Council of 23 November 2022. This regulation requires national authorities to share molecular pathogen data when necessary for the detection and investigation of serious cross-border health threats [4].

Beyond providing genomic typing and epidemiological data integrated with genomic analysis results to relevant stakeholders, the RKI already offers direct support to local and state public health authorities, facilitates supra-regional cluster detection, and serves as a rapid response hub for cross-state transmission events [5,6].

The establishment of this central national backbone is essential for detecting outbreaks at both national and international levels, while aligning with the LPHA-focused approach proposed by the authors. Furthermore, it meets the statutory responsibilities conferred upon the RKI by §§ 13 and 14 of the German Infection Protection Act (IfSG).

Bludau et al. highlight the importance of strong partnerships between LPHAs and university hospitals. While we fully agree with this idea, it is important to recognise the structural challenges involved, as not all LPHAs have the opportunity to engage in close collaboration with university hospitals that possess established expertise and infrastructure in pathogen genomics. Accordingly, support from state-level authorities is essential; where such support is constrained by limited resources, national-level assistance becomes indispensable. Moreover, it remains unclear how promptly bottom-up approaches will be able to contribute sequence data to the overarching surveillance systems – despite the fact that timeliness is critical for reducing case numbers and enabling effective outbreak control.

In summary, Bludau et al. provide an excellent and pragmatic blueprint for implementing integrated genomic surveillance at the LPHA level. By embedding this framework within a robust national infrastructure, outbreaks – whether local or multistate – can be detected early, analysed thoroughly and contained rapidly, regardless of their origin. This, in turn, can considerably reduce the burden of infectious diseases in the population in Germany and elsewhere.
